# Research note: Use of *in vitro* and *in ovo* approaches to characterise avian influenza viruses and assess species specific responses to infection

**DOI:** 10.1016/j.psj.2025.105987

**Published:** 2025-10-19

**Authors:** Lamyaa Al-Dalawi, Jiayun Yang, Adam M. Blanchard, Michael Clark, Kin-Chow Chang, Munir Iqbal, Leah V. Goulding

**Affiliations:** a School of Veterinary Medicine and Science, University of Nottingham, Nottingham, UK; b The Pirbright Institute, Pirbright, Woking, UK

**Keywords:** Avian influenza, In vitro, In ovo, Embryonated egg, Avian cell

## Abstract

Circulation of avian influenza viruses (AIVs) in domestic and wild birds poses a major health threat, requiring a rapid approach to characterize and identify high-risk strains. We evaluated virus replication kinetics and host viability with low pathogenicity avian influenza (LPAI) H6N1 and genetically modified avian influenza GM-AIV09 viruses in embryonated eggs and avian cell lines. Virus titres increased steadily in a 72-hour infection in DF-1 cells and embryonated hens’ eggs but peaked at 48 hours in CCL-141 cells and embryonated duck eggs. CCL-141 cells exhibited greater virus-induced cell death than DF-1 cells, whereas duck embryos maintained higher viability than chicken embryos. Additional work is needed to establish if there are breed specific differences which may impact categorisation of AIVs using embryonated eggs**.** Use of embryonated eggs better mirrored relative avian species survivability to AIV infection than use of avian cells.

## Introduction

Avian influenza viruses (AIVs), particularly subtypes such as H5N1, continue to pose a major threat to poultry health worldwide. These viruses can circulate in both domestic and wild bird populations, with varying clinical outcomes depending on the host species (Swayne, 2013). Ducks often remain asymptomatic carriers (Brauer and Chen, 2015; Webster et al., 1992), whereas chickens typically show more severe disease outcomes following infection, including increased mortality. The underlying mechanisms of species-specific differences in host susceptibility and immune responses to AIV infection continues to merit further research. Regular AIV outbreaks in domestic poultry and wild birds in the UK and elsewhere, highlight the need for tools that better capture species-specific responses and to enable rapid characterisation of emerging AIVs (de Bruin, et al., 2022).

This study compares AIV replication in chickens and ducks using *in vitro* (cell culture) and *in ovo* (embryonated egg) models, examining viral replication rates, cell viability, and embryo survival. We highlight how *in ovo* models are a viable alternative where appropriate cell lines are lacking and are an ethical alternative to *in vivo* models, in line with the 3Rs principle to avoid or replace the use of animals, to assess emerging AIV strains.

## Materials and methods

### Viruses

A LPAI H6N1 virus (A/turkey/England/198/09) and a recombinant PR8-H5N1 virus, which contains the internal six segments from highly pathogenic avian influenza virus A/chicken/Scotland/054477/2021 (H5N1), HA and NA from laboratory adapted A/Puerto Rico/8/1934 (H1N1), referred to as GM-AIV09, were used in this study. GM-AIV09 was generated by reverse genetics (RG) using an eight-plasmid bidirectional pHW2000 vector system as previously described (Neumann, et al., 1999). All viruses were propagated in the allantoic fluid of 10-day-old embryonated chicken eggs described previously (Brauer and Chen, 2015).

### Cells

DF-1 (chicken embryo fibroblast; ATCC CRL-12203) cells were maintained in DMEM-Glutamax with 5 % fetal calf serum (FCS), 5 % chicken embryo extract, and 100U/mL penicillin-streptomycin (P/S) (Gibco, ThermoFisher Scientific, Paisley, UK). and MDCK (Madin-Darby Canine Kidney) cell lines were maintained in DMEM with 10 % FCS and 100U/mL P/S.

### Cell culture and virus quantification

Cells were infected with the H6N1 or GM-AIV09 virus in OptiMEM medium (ThermoFisher Scientific, Waltham, Massachusetts, USA) containing 100 U/mL P/S and 125ng/mL TPCK trypsin (Sigma-Aldrich) at a multiplicity of infection (MOI) of 0.25. Supernatants were collected at 2, 24, 48 and 72 hours post infection (hpi) for focus forming assay (FFA) on MDCK cells, as described previously (Al-Beltagi, et al., 2021) and for quantitative real-time reverse transcription (RT-qPCR).

Viral RNA was extracted using the QIAamp Viral RNA Mini Kit (Qiagen) following the manufacturer’s instructions. RT-qPCR was performed using QuantiFast SYBR Green RT-PCR Kit (Qiagen) on a Roche LightCycler. Primers for the H6N1 virus M gene were sense 5ʹ- CGCGCAGAGACTTGAAGAT-3ʹ and antisense 5ʹ- CTTAGTCAGAGGTGACAGGATTG-3ʹ, and for the GM-AIV09 M gene were sense 5ʹ- GTTGGCCAGTACTACAGCTAAG-3ʹ and antisense 5ʹ- TCATCGCCTGCACCATTT-3ʹ. Viral gene expression was determined using the relative Ct method.

### Cell viability assay

DF-1 and CCL-141 cells were infected with LPAI H6N1 or GM-AIV09 virus at the indicated MOIs in six replicates and viability was assessed at 2, 24, 48 and 72 hpi using a MTT assay (CellTiter 96® Aqueous one solution for cell proliferation assay; Promega) per the manufacturer’s protocol.

### Embryonated egg infection

Embryonated Dekalb White hen (10 days) and pekin duck eggs (18 days) were inoculated with 100 µL of a 1:100 dilution (MOI of 1.25, determined by FFA on MDCK cells) of the H6N1 or GM-AIV09 viruses into the allantoic cavity. The eggs were sealed and incubated at 37.5°C and 45-50 % humidity. At each time point (2, 24, 48, and 72 hpi), a minimum of five eggs per group (*n* > 5/group) were inoculated and analysed across independent experimental replicates. Allantoic fluid was collected, centrifuged and stored at −80°C. For viral replication, 140 µL of the allantoic fluid was reserved for RNA extraction and the remainder for FFA (Section 2.3).

Embryo viability was monitored by candling (illuminating the egg to observe blood vessels and embryo movement). Live embryos displayed intact blood vessels and movement, dead embryos exhibited no movement and collapsed blood vessels, as described previously(Brauer and Chen, 2015). Survival was calculated using Kaplan-Meier survival analysis in GraphPad Prism 10.

## Results and discussion

This study evaluated *in vitro* and *in ovo* models, comparing duck and chicken cell lines and embryonated eggs, to assess host-specific responses to AIV infection. Although cell lines support replication studies, they lack species-specific complexity and are unavailable for many avian species. Here, we demonstrate the value of *in ovo* models for characterising emerging AIVs.

### Embryonated eggs better reflect relative in vivo survivability to AIV infection than the in vitro infection model

**Fig. 1:**
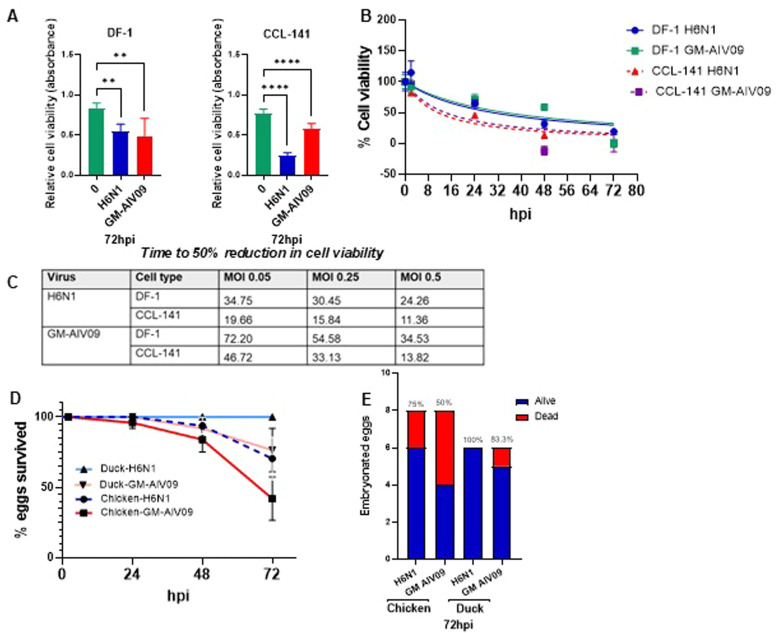
Duck embryonated eggs survived better than chicken embryonated eggs but chicken DF-1 cells survived better than duck CCL-141-cells infected with AIVs. (A) Cell viability of DF-1 and CCL-141 cell lines infected with H6N1 or GM-AIV09 at MOI of 0.25 at 72 hpi. (B) Representative regression curves of percentage cell viability, measured by MTT assays, of DF-1 and CCL-141 cells infected with H6N1 or GM-AIV09 virus at MOI of 0.5 for 2, 24, 48 and 72 hpi. Error bars represent the SEM. (C) Time (hours) required for a 50 % reduction in cell viability in DF-1 and CCL-141 cells infected at the MOI of 0.05, 0.25 and 0.5. 50 % reduction in cell viability determined by non-linear regression on normalised data. (D) Embryo survival in percentages (*n* > 5/ group) at 2, 24, 48 and 72 hpi with H6N1 or GM-AIV09 virus infection. (E) Percentage survival of chicken and duck embryonated eggs infected with H6N1 or GM-AIV09 at 72 hpi.

We first evaluated cell and embryonated egg survival following AIV infection. An initial comparison of cell viability at the end of a 72 h infection (at an MOI of 0.25), demonstrated a greater reduction in CCL-141 cells infected with the wild type H6N1 virus (68.6 %) than GM-AIV09 virus (28.1 %), showing greater susceptibility to H6N1 virus-induced cytopathic effects, while DF-1 cells displayed a similar reduction in cell viability to H6N1 virus (34.3 %) and GM-AIV09 virus (36.9 %) (Fig. 1A). To compare onset of cell death between cell types and virus strain, we determined the time taken for 50 % cell death following infection with AIV over the 72 h infection. Consistent with studies reporting rapid apoptosis in duck cells(Kuchipudi, et al., 2012), our MTT assay demonstrated earlier cell death in CCL-141 cells than in DF-1 cells, and this pattern was consistent for both H6N1 virus and GM-AIV09 virus infections (Fig. 1B&C).

However, chicken embryos were more susceptible to virus-induced mortality than duck embryos. 10-day old embryonated hens’ eggs and 18-day old embryonated duck eggs were infected with the indicated MOI of virus and viability measured every 24 hours. H6N1 virus infection reduced chicken embryo survival from 100 % at 2 hpi to 75 % by 72 hpi, while duck embryos remained at 100 % throughout. Similarly, GM-AIV09 infection reduced duck embryos survival to 80 % compared to 50 % in chicken embryos at 72 hpi (Fig. 1D). Fig. 1E compares survival 72 hpi, summarising the higher survival in duck than chicken embryos.

As ducks generally display greater resistance and survival rates to AIV infection than chickens *in vivo,* the *in ovo* model better reflects disease outcome across species.

Our results identified species specific variation in survival and AIV replication dynamics. Consistent with previous *in vitro* studies comparing apoptosis, duck cells exhibited faster cell death compared to chicken cells in a 72 h infection(Kuchipudi, Dunham, Nelli, White, Coward, Slomka, Brown and Chang, 2012). However, embryonated duck eggs showed higher survival rates than chicken eggs, in line with *in vivo* findings (Burggraaf, et al., 2014). This highlights the in ovo model’s potential value in predicting disease outcomes across species and to different virus strains. This complements previous studies emphasizing the importance of chicken embryo tissues to understand influenza virus replication and evolution (Seekings, et al., 2020).

### Chicken cells and embryonated eggs sustained AIV replication longer than duck cells and embryonated eggs

The replication kinetics of H6N1 and GM-AIV09 viruses were evaluated over a 72 h infection period and compared between species using two experimental systems: *in vitro* (DF-1 chicken and CCL-141 duck cell lines, infected at an MOI of 0.25) and *in ovo* (10-day old embryonated chicken eggs and 18-day old embryonated duck eggs, infected at an MOI of 1.25). At 2, 24, 48 and 72 hpi, total viral load in the cell culture supernatant and allantoic fluid was quantified by RT-qPCR and infectious titres were measured by FFA.

**Fig. 2:**
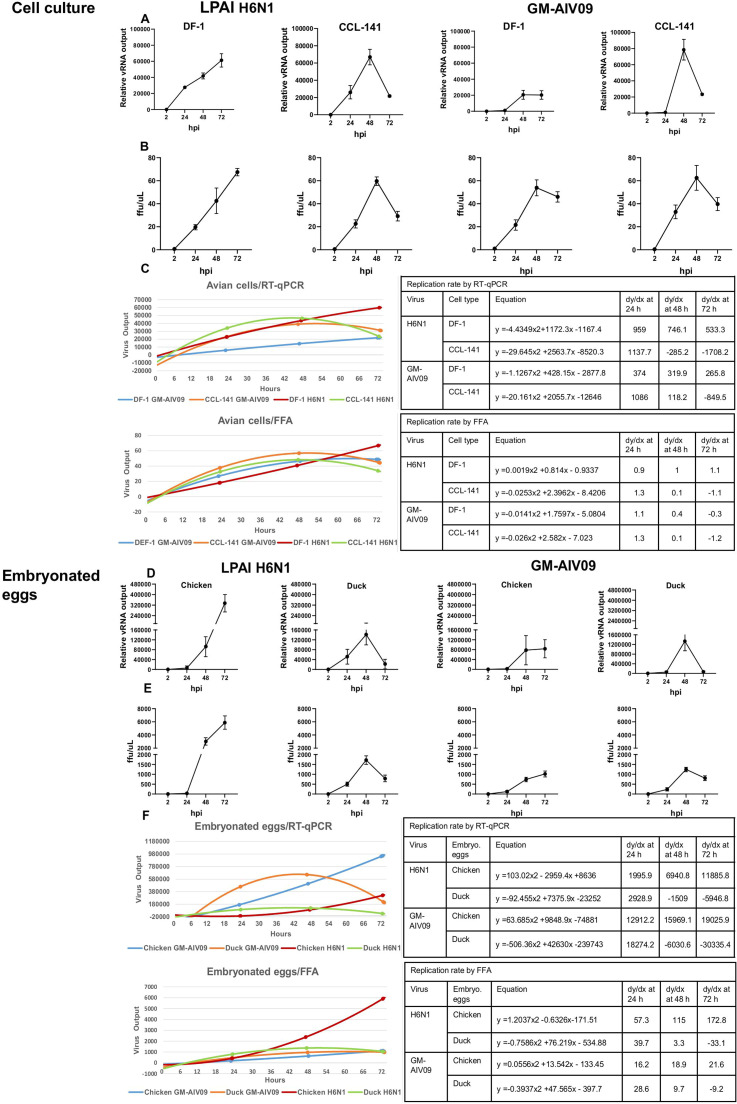
Chicken cells and embryonated eggs sustained AIV replication longer than corresponding duck cells and embryonated eggs. Replication dynamics of LPAI H6N1 virus and GM-AIV09 viruses in chicken DF-1 and duck CCL-141cells infected at 0.25 MOI (A to C), and in ovo in 10-day old embryonated Dekalb White hen eggs and 18-day old Pekin duck eggs (D to F) at the equivalent dose of 1.25 MOI in 0.1 ml/egg. Supernatants from infected cell cultures, and allantoic fluids (from ≥ 5 eggs per species) were collected at 2, 24, 48 and 72 hpi. Results are expressed as mean ± SD from three independent experiments: each experiment ≥ 3 technical replicates for cell cultures and ≥ 5 embryonated eggs for in ovo infections. Virus replication was quantified by RT-qPCR of viral M-gene RNA (A and D), and focus-forming assays for infectious virus output (B and E) expressed as FFU/µl. Growth curves and corresponding equations derived from viral RNA and FFU output over 72 hpi (C and F).

By directly comparing *in vitro* and *in ovo* approaches, we were able to evaluate how each model reflects the dynamics of AIV replication and to assess their suitability for characterizing virus strains. In both systems, replication kinetics differed by host species. H6N1 and GM-AIV09 virus titres increased over 72 hpi in DF-1 cells and chicken embryonated eggs, while replication peaked at 48 hpi in CCL-141 cells and duck embryonated eggs (Fig. 2A, B, D, E).

We quantified virus output changes over time (calculating the derivative, dy/dx) to calculate and compare relative replication rates. At 48 hpi, RT-qPCR derived replication rates were highest in DF-1 cells (H6N1: 746.1; GM-AIV09: 319.9) and chicken embryonated eggs (H6N1:6940.8; GM-AIV09:15969.1). While lower or negative values were observed in CCL-141(H6N1: −285.2; GM-AIV09:118.2) and embryonated duck eggs (H6N1: −1509, GM-AIV09: −6030.6) (Fig. 2C, F). Replication rates calculated from FFA data (as the derivative of infectious virus production over time) showed a similar trend: at 48 hpi, rates were higher in DF-1 cells (H6N1: 1; GM-AIV09: 0.4) and chicken embryonated eggs (H6N1: 115; GM-AIV09:18.9), compared to CCL-141 cells (both viruses: 0.1) and duck eggs (H6N1: 3.3; GM-AIV09: 9.7) (Fig. 2C,F). Due to its sensitivity and compatibility with high-throughput processing we recommend evaluating total progeny virus output by RT-qPCR and to use the *in ovo* model, which better reflects virus infection *in vivo*, for evaluating virus strains across species.

Virus replication rates also differed across species, reflected in both models. We observed consistent AIV production over 72 hours in the chicken embryonated eggs and cell line. In contrast, virus replication in duck embryonated eggs and cell cultures decreased after 48 hours, indicating a species-specific response. This approach could enhance understanding of tropism; previous studies show differences of the pathobiology of AIVs across avian species, with ducks often supporting milder infection (Kim, et al., 2009). The decrease in AIV replication after 48 hours may reflect previously reported stronger antiviral response in ducks, such as upregulation of key antiviral genes, capable of inhibiting virus replication (de Bruin, Spronken, Bestebroer, Fouchier and Richard, 2022).

Our study supports the use of *in ovo* models as a physiologically relevant system for AIV research, as they preserve host complexity and more accurately mimic infection dynamics in the avian host than cells lines, thereby providing valuable insight into species responses and ultimately may assist in poultry health management.

## Funding

This study was supported by the Biotechnology and Biological Sciences Research Council (BBSRC) [grant numbers BB/Y007298/1, BBS/E/PI/230002A], and the Medical Research Council (MRC) [grant number MR/Y03368X/1].

## CRediT authorship contribution statement

**Lamyaa Al-Dalawi:** Writing – review & editing, Writing – original draft, Resources, Methodology, Investigation, Formal analysis, Data curation. **Jiayun Yang:** Writing – review & editing, Methodology. **Adam M. Blanchard:** Writing – review & editing, Methodology. **Michael Clark:** Writing – review & editing, Methodology. **Kin-Chow Chang:** Writing – review & editing, Writing – original draft, Validation, Supervision, Software, Project administration, Methodology, Investigation, Formal analysis. **Munir Iqbal:** Writing – review & editing, Methodology. **Leah V. Goulding:** Writing – review & editing, Writing – original draft, Visualization, Validation, Software, Resources, Methodology, Investigation, Formal analysis, Data curation.

## Disclosures

The authors declare that they have no known competing financial interests or personal relationships that could have appeared to influence the work reported in this paper.
